# The Effect of Ethanol Treatment on the Quality of a New Table Grape Cultivar It 681–30 Stored at Low Temperature and after a 7-Day Shelf-Life Period at 20 °C: A Molecular Approach

**DOI:** 10.3390/ijms22158138

**Published:** 2021-07-29

**Authors:** Irene Romero, Maria Vazquez-Hernandez, Manuel Tornel, M. Isabel Escribano, Carmen Merodio, M. Teresa Sanchez-Ballesta

**Affiliations:** 1 Department of Characterization, Quality and Safety, Institute of Food Science, Technology and Nutrition, Spanish National Research Council (ICTAN-CSIC), José Antonio Novais 10, E-28040 Madrid, Spain; irene.romero@ictan.csic.es (I.R.); mavahe86@gmail.com (M.V.-H.); escribano@ictan.csic.es (M.I.E.); merodio@ictan.csic.es (C.M.); 2 Instituto Murciano de Investigación y Desarrollo Agrario y Medioambiental (IMIDA), Mayor, s/n, La Alberca, E-30150 Murcia, Spain; manuel.tornel@carm.es

**Keywords:** table grapes, ethanol, low temperature, fruit quality, gene expression

## Abstract

Despite the fact that many studies have examined the effectiveness of different gaseous postharvest treatments applied at low temperature to maintain table grape quality, the use of ethanol vapor has hardly been investigated. Thus, this work has studied the effectiveness of ethanol vapor-generating sachets in the maintenance of It 681–30 table grape quality, a new cultivar, during storage at low temperature and after the shelf-life period at 20 °C. To this end, various quality assessments have been carried out and the effect of the ethanol treatment on the expression of different genes (*phenylpropanoids, transcription factors, PRs*, and *aquaporins*) was determined. The results indicated that the application of ethanol vapor reduced the total decay incidence, weight loss, and the rachis browning index in It 681–30 grapes stored at 0 °C and after the shelf-life period at 20 °C, as compared to non-treated samples. Moreover, the modulation of *STS7* and the different *PR* genes analyzed seems to play a part in the molecular mechanisms activated to cope with fungal attacks during the postharvest of It 681–30 grapes, and particularly during the shelf-life period at 20 °C. Furthermore, the expression of *aquaporin* transcripts was activated in samples showing higher weight loss. Although further work is needed to elucidate the role of ethanol in table grape quality, the results obtained in this work provide new insight into the transcriptional regulation triggered by ethanol treatment.

## 1. Introduction

Table grape is a non-climacteric fruit, subject to water loss and decay during postharvest handling. The storage of table grapes at low temperature (around 0 °C), with high relative humidity, is one of the most widely used technologies for maintaining their postharvest quality. However, this is normally not sufficient to avoid senescence of the rachis, abscission of berries, or a fungal attack, mainly caused by *Botrytis cinerea*, all of which compromise bunch quality (reviewed by [1]). Thus, different postharvest treatments have been applied alongside low-temperature storage to maintain table grape quality, the most used being the modification of the storage atmosphere by increasing the O_2_ or CO_2_ concentration [2,3,4,5,6,7,8]. Among these treatments, the use of ethanol vapor has been less studied so far. Ethanol is considered to be a “generally recognized as safe” (GRAS) compound [9], and as such, it can be used in the food industry. It has been found that the application of ethanol effectively reduces fungal attack and delays senescence in fruit and vegetables [9,10,11,12,13,14]. The response of fruit to ethanol depends on different factors, such as the species, cultivar, and maturity, together with the dose and duration of exposure [10]. In the particular case of table grapes, it is known that ethanol applied as vapor or in solution limits *Botrytis* development over the postharvest period, maintaining the organoleptic quality of berries [15,16,17]. However, while the use of ethanol vapor-generating sachets, such as Antimold^®^, controlled fungal growth [18] and increased the anthocyanin content and antioxidant capacity during storage, it also adversely affected the rachis, inducing browning in Red Globe bunches [19]. It is important to highlight that, to date, most published research reported on the effectiveness of gaseous treatments in table grapes refers to increasing the concentration of O_2_ or CO_2_ in the storage atmosphere [1]. Likewise, most studies related to the application of ethanol in table grapes have addressed the effect on bunch quality, but there is still a dearth of knowledge about the mechanisms involved in the effectiveness of this treatment. Consequently, in order to form the basis of knowledge about the effectiveness of ethanol treatments, an in-depth study needs to be conducted of the molecular mechanisms related to their effectiveness, which are currently unknown. 

The activation of phenylpropanoid metabolism plays a role in the response of plants to abiotic stress, as well as in the defense against pathogens [20,21]. In the case of table grapes, the expression of phenylpropanoid pathway genes has been studied in response to different postharvest treatments. By way of example, applying 5 kPa O_2_ and 15 kPa CO_2_ for 6 weeks at 0 °C activated the expression of 13 *PAL* and 6 *STS* transcripts in Superior Seedless grapes [8]. Furthermore, short-term treatment with high levels of CO_2_ activated the accumulation of *CHS* transcripts in Autumn Royal table grapes [22] and white Dominga table grapes. Additionally, there was an increase in the accumulation of stilbene compounds in CO_2_-treated samples, which seems to be modulated by *VviSTS6*, *VviSTS7*, and *VviSTS46* [23]. 

Previous research has reported that pathogenesis-related proteins (PRs) seem to have a protective role in table grapes during postharvest. Applying a postharvest treatment with SO_2_ or O_3_ delayed fungal growth in Red Globe grapes and increased the accumulation of *chitinase* and *β-1,3-glucanase* transcripts [24]. Likewise, recombinant class I chitinase and β-1,3-glucanase from table grapes showed in vitro cryoprotective and antifungal activities [25,26]. Moreover, Crimson Seedless lines expressing cisgenic thaumatin-like proteins [27] or chitinase and β-1,3-glucanase proteins [28] displayed resistance to powdery or downy mildew, respectively. 

On the other hand, it is known that in plants, water transport across the membranes is facilitated by the water channel proteins called aquaporins [29]. In addition to water, these proteins facilitate the transport of small neutral solutes and gases [29]. The expression of aquaporin genes decreased in response to water stress in grapes [30] and to low temperature in Arabidopsis [31]. Furthermore, the heterologous expression of *PIP1;1* from banana in Arabidopsis confers tolerance to water and salt stress by reducing membrane damage, improving ion distribution and maintaining the osmotic balance [32]. Considering that there is limited information about the role of aquaporins in table grapes during postharvest, its analysis could be helpful to study the effectiveness of postharvest treatments in table grape water status. 

In recent years, researchers and companies have focused on breeding new table grape cultivars, resulting in increased growth and profitability for retailers and growers around the world. Hence, it is imperative that these advances are accompanied by the study of the postharvest behavior of these new cultivars. The cultivar It 681–30 is the result of crossbreeding ((Dominga × Moscatuel) × Crimson), obtained in a table grape breeding program developed in the region of Murcia (Spain) by the Table Grape Research and Technology Society, ITUM, in collaboration with the Murcia Institute for Agricultural and Food Research and Development (IMIDA). It 681–30 is a late-harvesting cultivar, with a harvest period from mid-September to the end of November. It 681–30 berries are seedless, elliptical in shape, and with a size that varies naturally between 17 and 19 mm, while after treatment with gibberellic acid and girdling, it reaches 22–24 mm. The pulp is juicy, the acidity taste is neutral, and it has a crunchy texture. However, the mechanisms modulated by applying postharvest treatments such as ethanol are, so far, unknown for this new cultivar. Thus, this study aimed to explore, on the one hand, the postharvest behavior of the new It 681–30 cultivar during storage at low temperature, and on the other hand, the molecular mechanisms linked to the effectiveness of ethanol vapor-generating sachets in the maintenance of the It 681–30 table grapes quality. To this end, different quality parameters have been analyzed in ethanol-treated and non-treated It 681–30 table grapes stored at 0 °C for up to 49 days and after a 7-day shelf-life period at 20 °C. Moreover, the expression of genes that encoded PRs (*Vcchit1b*, *Vcgns1, VviTL*, and *VviOsmo1*), enzymes (*VviPAL*, *VviCHS*, and *VviSTS7*), and transcription factors (*VviMYB13*, *VviMYB14,* and *VviMYB137*) related to phenylpropanoid biosynthesis and aquaporins (*VviPIP1.2*, *VviPIP1.3*, *VviPIP2.1*, and *VviPIP2.2*) were studied, for the first time, in ethanol-treated and non-treated table grapes. 

## 2. Results and Discussion

### 2.1. The Effect of an Ethanol Treatment on Quality of It 681–30 Bunches Stored at Low Temperature and during the Shelf-Life Period at 20 °C

**Figure 1:**
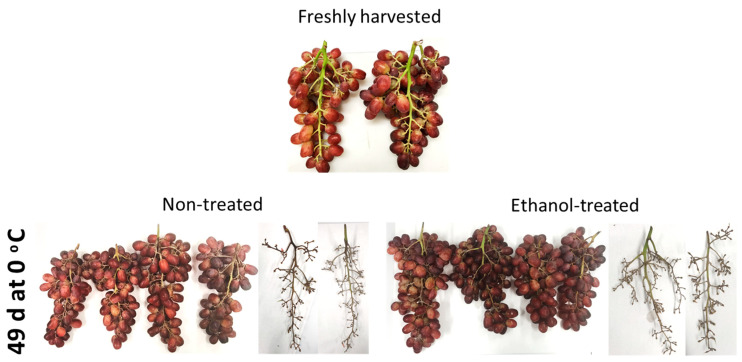
Appearance of It 680–31 grape bunches treated and non-treated with ethanol stored at 0 °C for up to 49 days.

In the present study, the postharvest response of the cultivar It 681–30 in low-temperature storage has been analyzed, as well as how the treatment of ethanol vapor-generating sachets can help improve the quality of these table grapes stored at 0 °C and after 7 days at 20 °C (Figure 1). The results indicated that SSC increased slightly both in non-treated and ethanol-treated grapes stored at 0 °C during the period at 20 °C (Table 1). However, TA and pH did not vary in any condition assayed. Low-temperature storage increased the weight loss in non-treated and ethanol-treated bunches, with this being significantly higher in those bunches stored in air. Moreover, the shelf-life period increased the weight loss, although the percentage of this loss was lower in the ethanol-treated bunches. The total decay was significantly lower in the ethanol-treated table grapes. Furthermore, although the shelf-life period of 7 days at 20 °C significantly increased total decay in both ethanol-treated and non-treated It 681–30 samples, the values were significantly lower in those that had been treated (Table 1). Regarding the rachis-browning index, only ethanol-treated bunches stored at 0 °C showed significantly lower values. The results of this study are in concordance with previous works, where the application of ethanol vapor treatments controlled rot development [16]. These authors also observed that rachis browning was lower in ethanol-treated grapes in comparison to the control fruit but not when SO_2_-treatment was used. However, Lurie et al. [17] observed that rachis desiccation was similar in control, ethanol-treated, and SO_2_-treated Thompson Seedless table grapes. The effect of ethanol on rachis browning seems to be cultivar-dependent. Thus, Candir et al. [33] indicated that packing Pafi grapes with Antimold^®^60 sachets resulted in lower weight loss and found no adverse effects as to rachis browning, TA, and SSC after 3 months of storage at 0 °C. 

### 2.2. The Effect of Storage at 0 °C and the Shelf-Life Period at 20 °C on the Phenylpropanoid Gene Expression in the Skin of Non-Treated and Ethanol-Treated It 681–30 Table Grapes

Although the beneficial effects of ethanol treatments on table grape quality during postharvest have been reported [17,18,19,33], to our knowledge, the molecular mechanisms involved in maintaining fruit quality are not yet known. Grapes accumulate a number of secondary metabolites, including flavonoids and stilbenoids, whose synthesis and accumulation are affected by different postharvest conditions [8,22,23,34]. The expression of two (*VviPAL* and *VviCHS*) flavonoid pathway genes as well as one (*VviSTS7*) and three regulatory (*VviMYB14*, *VviMYB15A*, and *VviMYB15C*) stilbene pathway genes during the storage of non-treated and ethanol-treated It 681–30 table grapes at 0 °C and after 7 days at 20 °C were analyzed. 

#### 2.2.1. Expression of *VviPAL*, *VviCHS*, and *VviSTS7*

**Figure 2:**
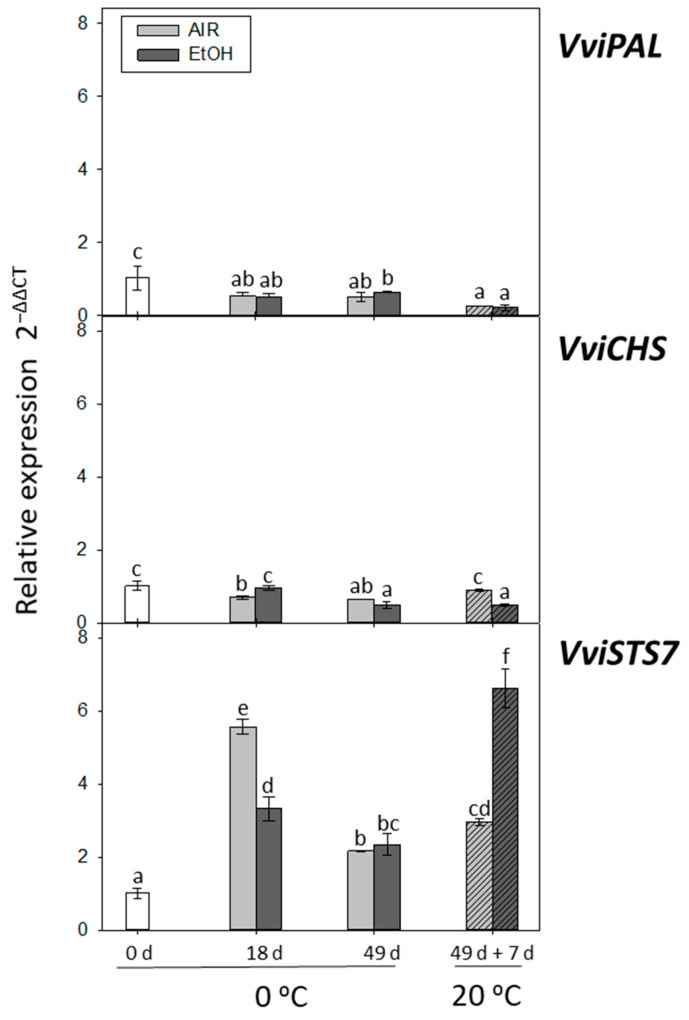
Effect of low temperature and ethanol treatment on *VviPAL, VviCHS*, and *VviSTS7* gene expression in the skin of It 681–30 table grapes stored for 49 days at 0 °C and after the shelf-life period (49 d + 7 d at 20 °C). The transcript levels of each gene were assessed by RT-qPCR and normalized using *Actin1* as a reference gene. The results were calculated relative to a calibrator sample (time 0) using the formula 2^−ΔΔCt^. Values are the mean ± SD, *n* = 6. Different letters on bars indicate that the means are statistically different using the Tukey-b test (*p* < 0.05).

The gene expression of *VviPAL* decreased over the course of the storage period at 0 °C and after 7 days at 20 °C, both in the skin of non-treated and ethanol-treated table grapes (Figure 2). However, a decrease of *VviCHS* gene expression was also denoted in non-treated grapes stored at 0 °C. In the case of ethanol-treated samples, the decrease was not noted until day 49 at 0 °C (Figure 2). At 20 °C, the levels of *VviCHS* only increased in non-treated grapes, reaching values similar to freshly harvested fruit (Figure 2). These gene expression patterns showed a significant positive correlation (*r* = 0.451, *p* < 0.05) (Table 2). While no works have reported the modulation of these genes in ethanol-treated table grapes during postharvest, what has been previously studied is the effect of ethanol on the phenylpropanoid gene expression in Cabernet Sauvignon berries during their development after *veraison* [35]. These authors observed that spraying grapes at *veraison* with ethanol at 5% in water had no stimulating effect on the transcription of *CHS*, *DFR*, *F3H,* and *LDOX* genes. Furthermore, ethanol treatment was able to inhibit phenolic metabolism, which is critical for tissue browning in lettuce, by repressing the expression of *PAL* mRNA and inhibiting PAL activity [36]. 

On the other hand, *VviSTS7* gene expression showed a sharp and transient increase at day 18, both in the skin of ethanol-treated and non-treated It 681–30 grapes, although it was higher in non-treated samples (Figure 2). At day 49, transcript accumulation decreased in both samples, decreased to similar levels, which were higher than those recorded for freshly harvested grapes. After the shelf-life period at 20 °C, *VviSTS7* gene expression increased in both treated and non-treated table grapes. Yet, the ethanol-treated grapes showed values seven times higher than the freshly harvested ones and twice the levels observed in non-treated grapes. *VviSTS7* gene expression showed a significant negative correlation with the transcript accumulation of *VviPAL* (*r* = −0.577, *p* < 0.01) and *VviCHS* (*r* = −0.4401, *p* < 0.05) (Table 2). It is well known that the application of different postharvest treatments, such as UV-B, UV-C, and high levels of CO_2_, which improve the quality of table grapes, also activate the expression of *STS* genes and the accumulation of resveratrol [8,23,37]. Nonetheless, this is the first study demonstrating the modulation of *STS* genes by ethanol treatment. Overexpression of these genes has been previously reported to improve resistance against fungal pathogens [38,39]. The fact that *STS7* gene expression increases sharply in ethanol-treated samples after the shelf-life period, where the total decay is 1.83 times lower than in non-treated fruit, might be part of the molecular strategy of these grapes to cope with fungal attacks.

#### 2.2.2. Expression of *VviMYB13*, *VviMYB14*, and *VviMYB137*

**Figure 3:**
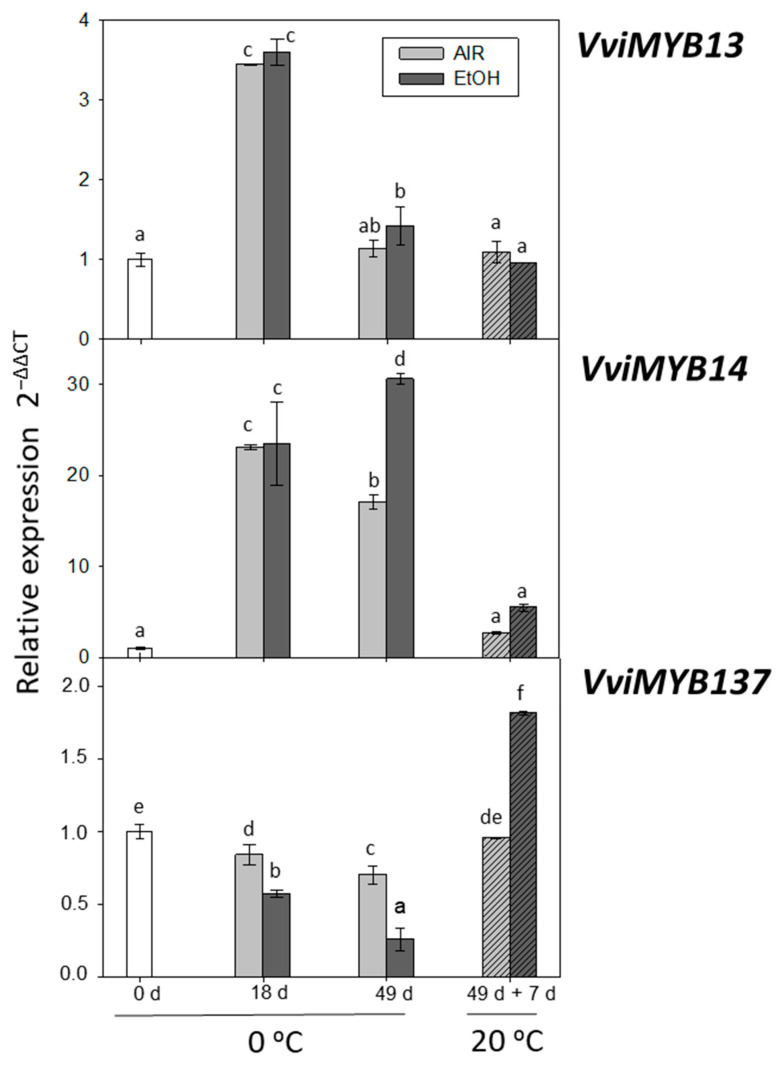
Effect of low temperature and ethanol on *MYB14, MYB15A*, and *MYB15C* transcription factors gene expression in the skin of It 681–30 table grapes stored for 49 days at 0 °C and after the shelf-life period (49 d + 7 d at 20 °C). The transcript levels of each gene were assessed by RT-qPCR and normalized using *Actin1* as a reference gene. The results were calculated relative to a calibrator sample (time 0) using the formula 2^−ΔΔCt^. Values are the mean ± SD, *n* = 6. Different letters on bars indicate that the means are statistically different using the Tukey-b test (*p* < 0.05).

*MYB* transcription factors are known to play key roles in regulating the phenylpropanoid pathway [40]. Previous studies have reported [8,41] that the expression of *MYBs* can be modulated through storage at low temperature or by applying gaseous postharvest treatments. However, as of yet, their response to ethanol treatment has not been studied. The results of this study indicated that gene expression of *VviMYB13* and *VviMYB14* showed a sharp increase at day 18 in the skin of both treated and non-treated samples (Figure 3). It was, nevertheless, transitory in the case of *VviMYB13*, reaching levels similar to freshly harvested grapes at day 49 and after the shelf-life period. By contrast, the increase in *VviMYB14* transcript accumulation at 0 °C also took place at day 49 in both samples, although this increase was higher in the ethanol-treated samples, decreasing after the time stored at 20 °C to levels similar to freshly harvested grapes. The *VviMYB137* transcript accumulation decreased during the storage at 0 °C in the skin of both ethanol-treated and non-treated grapes. However, the *VviMYB137* gene expression rose at the end of the shelf-life period at 20 °C in ethanol-treated samples (Figure 3).

Regarding the correlation between the expression of the three *VviMYBs*, the results showed that it was significantly positive between *VviMYB13* and *VviMYB14* (*r* = 0.584, *p* < 0.01). Conversely, *VviMYB137* gene expression was negatively correlated solely with *VviMYB14* (*r* = −0.701, *p* < 0.01). According to Vannozzi et al. [42] and Tyagi et al. [43], *VviMYB13*, *VviMYB14*, and *VviMYB137* could be involved in stilbene biosynthesis. However, according to the results of this study, only the *VviMYB137* transcript accumulation showed a significant positive correlation with *VviSTS7* gene expression (*r* = 0.607, *p* < 0.01) in response to low temperature and ethanol-treatment in It 681–30 grapes stored at 0 °C and during the shelf-life at 20 °C (Table 2). 

### 2.3. The Effect of Storage at 0 °C and the Shelf-Life Period at 20 °C on the Total Phenolic and Anthocyanin Content and the Antioxidant Capacity in the Skin of Non-Treated and Ethanol-Treated It 681–30 Table Grapes

**Figure 4:**
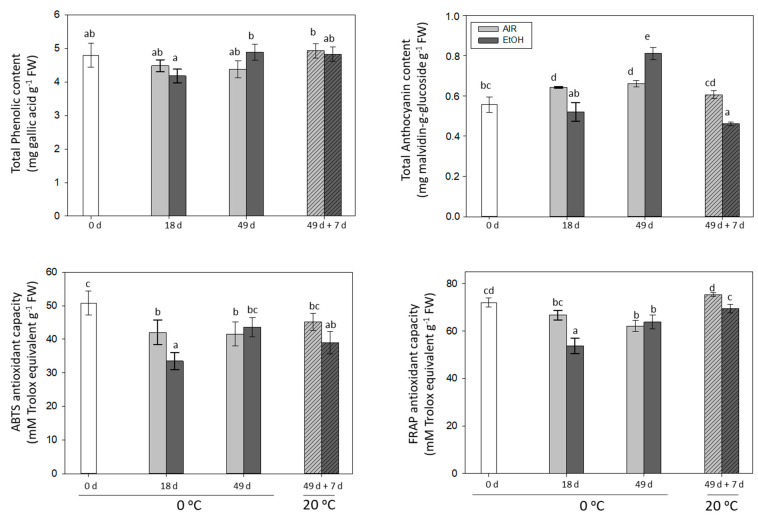
Changes in total phenolic content, anthocyanin content and antioxidant activity, determined by ABTS and FRAP, in the skin of non-treated and ethanol-treated It 681–30 table grapes stored up to 49 days at 0 °C and during the shelf-life period (49 d + 7 d at 20 °C). Values are the mean ± SD, *n* = 3. Different letters on the bars indicate that the values are statistically different using the Tukey-b test (*p* < 0.05).

The total phenolic levels remained constant during storage in all the samples analyzed, while the total anthocyanin content increased statistically significantly in non- treated samples stored at 0 °C, decreasing after 7 days at 20 °C to similar values to those achieved in freshly harvested grapes (Figure 4). Nonetheless, a significant increase was observed in ethanol-treated samples at the end of storage at 0 °C. Interestingly, no increase occurred during the shelf-life at 20 °C, where a significant reduction was observed in the ethanol-treated samples in comparison with non-treated and freshly harvested grapes. Although it is already known that phenolic and anthocyanin synthesis can continue after harvest and also during low-temperature storage, it is not a common response in grapes during postharvest. A decrease of the total anthocyanin content and maintenance of total flavonol content was found in Napoleon table grapes stored at low temperature [44]. Furthermore, Valero et al. [45] indicated a decrease in the total anthocyanin content of Autumn Royal grapes, as well as a significant loss of total phenolics during postharvest cold storage. Similar results were observed in three-day CO_2_-treated and non-treated Autumn Royal grapes stored at 0 °C [22]. Concerning the ethanol treatment, the results presented in this work are in concordance with Ustun et al. [19], who reported that postharvest ethanol treatment with Antimold^®^80 and Antimold^®^60 sachets increased the anthocyanin content of Red Globe grapes at low temperature. Additionally, ethanol treatment of Cabernet Sauvignon at *veraison* increased anthocyanin levels during ripening [35].

The antioxidant capacities, which were determined by the ABTS and FRAP methods, presented similar trends (Figure 4). Indeed, both methods revealed a decrease in the antioxidant capacities of It 681–30 table grapes during storage at 0 °C, showing only significant differences at day 18, when the ethanol-treated samples showed less antioxidant capacity than non-treated samples. Nevertheless, during shelf-life at 20 °C, while the values recorded by the ABTS method were similar to those observed at the end of storage at 0 °C, those achieved by the FRAP method were significantly higher. A significant positive correlation (*r* = 0.765, *p* < 0.01) was found between the results obtained by both methods measuring the antioxidant capacity of the grapes and also between total phenolic content and antioxidant activities (FRAP: *r* = 0.785, *p* < 0.01; ABTS: *r* = 0.735, *p* < 0.01). By contrast, the total anthocyanin content did not correlate with either total phenolic content or antioxidant activities. In Red Globe table grapes, FRAP and TEAC values rose with the increase in anthocyanin content activated by the treatment with Antimold^®^ sachets [19]. 

In this regard, it should be pointed out that different works studying the correlations between phenolic and anthocyanin compounds and the antioxidant capacity of grapes have shown inconsistent results. Thus, when applying one [46] of two [22] short-term CO_2_ treatments to table grapes, a positive correlation was obtained between their antioxidant capacity and changes in total phenolic content, but this was not the case in the pattern of total anthocyanins. Furthermore, total phenolic, flavonoids, and flavan-3-ols contents showed a positive correlation with the antioxidant capacity in different grape cultivars [47]. By contrast, when trans-resveratrol or glycine betaine was applied to maintain table grape quality during postharvest, no significant correlation was found between the antioxidant capacity changes and the total phenol and flavonoid levels [48]. 

### 2.4. The Effect of Storage at 0 °C and the Shelf-Life Period at 20 °C on PRs Gene Expression in the Skin and Pulp of Non-Treated and Ethanol-Treated It 681–30 Table Grapes

**Figure 5:**
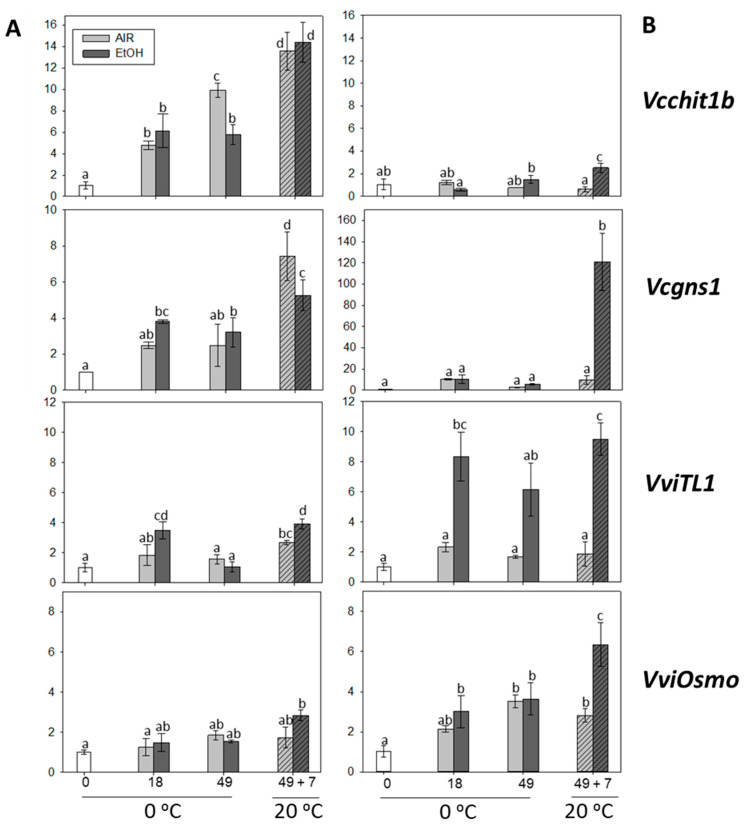
Effect of low temperature and ethanol treatment on *PRs* gene expression (*Vcchit1b, Vcgns1, VviTL1*, and *VviOsmo*) in the skin (**A**) and pulp (**B**) of It 681–30 table grapes stored for 49 days at 0 °C and after the shelf-life period (49 d + 7 d at 20 °C). The transcript levels of each gene were assessed by RT-qPCR and normalized using *Actin1* as a reference gene. The results were calculated relative to a calibrator sample (time 0) using the formula 2^−ΔΔCt^. Values are the mean ± SD, *n* = 6. Different letters on bars indicate that the means are statistically different using the Tukey-b test (*p* < 0.05).

Among the PR proteins, chitinase and β-1,3-glucanase play an essential role in plant defense mechanisms against biotic and abiotic stresses. Both enzymes are known to be able to hydrolyze polymers of fungal cell walls, and in combination, they can inhibit the growth of several pathogenic fungi in vitro [49]. This work analyzed the expression of genes that codified for class I chitinase (Vcchit1b), class I β-1,3-glucanase (Vcgns1), thaumatin (VvTL1), and osmotin (VviOsmo) in the skin and pulp of ethanol-treated and non-treated It 681–30 bunches, stored at 0 °C and after 7 days at 20 °C. The results showed that the expression of *Vcchit1b* and *Vcgns1* increased in the skin without significant differences between ethanol-treated and non-treated samples stored at 0 °C, except for *Vcchit1b* on day 49, whose expression was higher in non-treated samples (Figure 5A). Another finding was that the accumulation of both transcripts continued increasing after 7 days at 20 °C, only showing significant differences in the case of *Vcgns1*, whose expression was higher in the non-treated samples. By contrast, the expression of both genes did not change in the pulp of treated and non-treated grapes stored at 0 °C (Figure 5B). Meanwhile, the shelf-life period sharply activated their expressions in ethanol-treated grapes.

The *VviTL1* transcript levels only increased in the skin of ethanol-treated bunches after 18 days of storage at 0 °C (Figure 5A). Nevertheless, *VviTL1* gene expression was activated in both treated and non-treated samples during the shelf-life period and was higher in the treated grapes. Moreover, in the case of the pulp, a sharp increase in *VviTL1* accumulation was recorded independently of the temperature and time of storage in ethanol-treated samples (Figure 5B). On the other hand, storage at 0 °C did not change *VviOsmo* gene expression in the skin of either ethanol-treated or non-treated fruit, whereas the shelf-life activated its accumulation in ethanol-treated grapes. The gene expression increased in the pulp at day 49 at 0 °C and after 7 days at 20 °C in both samples, being significantly higher in the ethanol-treated ones after the shelf-life period (Figure 5B). 

It is also interesting to note that a positive and significant correlation was found in the expression of the four *PR* genes in the skin and the pulp (Table 3). The four *PR* genes analyzed showed a higher expression after the shelf-life period in ethanol-treated grapes, at which point the total decay of ethanol-treated fruit was 1.83 times lower than in non-treated fruit. Other studies have shown that in loquat fruit, treatment with ethanol induced the activities of chitinase and β-1,3-glucanase, which was accompanied by a lower disease incidence of anthracnose rot in ethanol-treated loquat fruit [50]. Furthermore, in line with these results, the inhibition of anthracnose rot development in tomato appears to be related to the positive impact of ethanol vapor on host resistance [51]. Hence, it seems that the modulation of these defense-related genes could play a role in the molecular mechanisms activated by It 681–30 grapes to cope with fungal attacks during postharvest. 

### 2.5. The Effect of Storage at 0 °C and the Shelf-Life Period at 20 °C on Aquaporins Gene Expression in the Skin and Pulp of Non-Treated and Ethanol-Treated It 681–30 Table Grapes

The effect of ethanol treatment on the expression of four aquaporin genes, which are considered factors that contribute to water loss, was analyzed. Thus, four genes encoding intrinsic plasma membrane proteins (*PIP1.2, PIP1.3, PIP2.1*, and *PIP2.2*) were examined in the skin and pulp of ethanol-treated and non-treated It 681–30 bunches stored at 0 °C and during shelf-life (49 d + 7 d).

**Figure 6:**
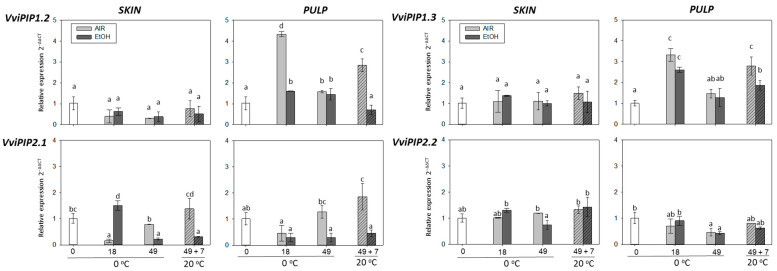
Effect of low temperature and ethanol treatment on *aquaporins* gene expression (*PIP1.2, PIP1.3, PIP2.1*, and *PIP2.2*) in the skin and pulp of It 681–30 table grapes stored for 49 days at 0 °C and after the shelf-life period (49 d + 7 d at 20 °C). The transcript levels of each gene were assessed by RT-qPCR and normalized using *Actin1* as a reference gene. The results were calculated relative to a calibrator sample (time 0) using the formula 2^−ΔΔCt^. Values are the mean ± SD, *n* = 6. Different letters on bars indicate that the means are statistically different using the Tukey-b test (*p* < 0.05).

In the skin, *PIP1.2*, *PIP1.3*, and *PIP2.2* gene expression did not change during storage at 0 °C or 20 °C (Figure 6). *PIP2.1* presented a significant and transient increase in ethanol-treated grapes after 18 days of storage at 0 °C, whereas *PIP2.1* transcript accumulation was significantly higher in non-treated samples at the end of storage at 0 °C and after 7 days at 20 °C. The gene expression of these *aquaporins* correlated significantly, with the exception of *PIP1.2* with *PIP2.2* (Table 4). The *PIP2.2* transcript levels in the pulp did not change over the storage period, while the *PIP1.2* and *PIP1.3* levels significantly increased both after 18 days and during shelf-life in non-treated samples. *PIP2.1,* for its part, showed a delay in the increase that was significant at the end of storage and was maintained during shelf-life. This study could only establish a significant correlation between *PIP1.2* and *PIP1.3* gene expression (*r* = 0.81, *p* < 0.01) (Table 4).

Some authors have suggested that *aquaporins*, especially *PIPs*, contribute, to a lesser extent, to water transport when the fruit cuticle presents microcracks [52]. In tomato, an enhanced expression of *PIP aquaporin* genes linked to increased water loss was reported in melatonin-treated grapes stored at 15 °C [53]. In strawberry, *PIP1* and *PIP2* gene expression increased coincident with a decrease in firmness during ripening, indicating that the reduction of fruit turgor together with the induction of *aquaporins* may accelerate the water outflow from cells [54]. Miranda et al. [55] provided evidence for a significant reduction in water loss linked to the down-regulation of two *PIP* genes in two cultivars of sweet cherries treated with melatonin. In this sense, the results showed that the highest increment in *PIPs* gene expression in It 681–30 grapes took place in non-treated fruit that exhibited the highest water loss. Similar results were found in Cardinal tables grapes, where an increase in *PIPs* gene expression was found in grapes stored at 0 °C [41]. 

## 3. Material and Methods

### 3.1. Plant Material and Storage Conditions

It 681–30 table grapes (*Vitis vinifera* L.) were collected in Abarán, Murcia, Spain (latitude: 38°12′00″ N; longitude: 01°24′00″ W; altitude 173 m) at optimum maturity (19.2% total soluble solids, 0.37% tartaric acid) in November 2018. Bunches were transferred to the ICTAN in Madrid (Spain) the same day of collection and those that did not present mechanical or pathological defects were randomly divided into two lots, each consisting of nine perforated polyethylene bags with four bunches per bag (about 3 kg). One batch was stored under normal atmospheric conditions (non-treated) for 49 days at 0 ± 0.5 °C with a relative humidity of 95%. The other batch was stored in the presence of two 6-g ethanol pads per bag (Antimold^®^60, Freund Industrial Co., Ltd., Tokyo, Japan) and stored at the same conditions of non-treated fruit. The ethanol pad allowed the ethanol vapor to diffuse gradually. The Antimold^®^ sachets are heat-sealed and are made of a laminated layer of paper and ethyl vinyl acetate copolymer, and contain microencapsulated food grade ethanol (58% by weight) absorbed onto silicon dioxide powder (35%). The encapsulated ethanol is released when in contact with water vapor. After the storage at 0 °C, both ethanol-treated and non-treated fruit were removed from the perforated polyethylene bags and transferred to boxes at 20 °C and stored for 7 days to simulate commercial shelf-life conditions. Eight bunches (approximately 750 g each bunch) were sampled at different time points and the skin and pulp were frozen in liquid nitrogen and stored at –80 °C until further analysis.

### 3.2. Quality Assessments 

Soluble solids content (SSC), titratable acidity (TA), and pH were determined in ethanol-treated and non-treated samples at day 0 and at day 49 of storage at 0 °C and after the shelf-life of 7 days at 20 °C, as described by Sanchez-Ballesta et al. [3]. Bunch weight was recorded on the day of harvest and after 49 days at 0 °C and 7 days at 20 °C. Cumulative weight losses were expressed as a percentage loss of the original weight. Total decay was expressed as the percentage of decayed berries at day 49 and after 7 days at 20 °C with respect to the original bunch weight. Rachis browning was determined by using the subjective scale as described by Vazquez-Hernandez et al. [56].

### 3.3. Relative Gene Expression by Quantitative Real-Time RT-PCR (RT-qPCR)

Total RNA extraction and cDNA synthesis were performed according to Romero et al. [57]. Relative expression of *PRs* (*Vcchit1b*, *Vcgns1, VviTL*, and *VviOsmo1*), *phenylpropanoid* (*VviPAL*, *VviCHS*, and *VviSTS7*), *transcription factor* (*VviMYB13*, *VviMYB14*, and *VviMYB137*), and *aquaporin* (*VviPIP1.2*, *VviPIP1.3*, *VviPIP2.1*, and *VviPIP2.2*) genes were studied in the skin and pulp of non-treated and ethanol-treated grapes stored at 0 °C for up to 49 days and after 7 days at 20 °C by RT-qPCR as described by Rosales et at [58]. Gene-specific primers were designed using Primer 3 software [59] and used to amplify specific products (Supplementary Table S1). *Actin1* (XM 002282480) from *V. vinifera* was used as the internal control (*Fw_Act1*: CTTGCATCCCTCAGCACCTT, *Rv_Act1*: TCCTGTGGACAATGGATGGA). The specificity of products was validated according to Romero et al. [57]. Three biological replicates and two technical replicates were performed for each sample.

### 3.4. Analysis of Total Anthocyanin Content

The determination of the total anthocyanin content in the skin of It 681–30 table grapes was carried out as described by Sanchez-Ballesta et al. [60]. For the extraction of total anthocyanins, 0.25 g of skin tissue from non-treated and ethanol-treated table grapes were homogenized with 0.75 mL of methanol (1% HCl acidified) using ultra sonication in cold water for 10 min. The extracts were centrifuged in cold at 10,000× *g* for 10 min and supernatants were collected. The previous steps were repeated until a volume of 1.5 mL was obtained. Samples were filtered with 0.45 μm nylon filters and stored at −80 °C. During the extraction, tubes were kept in the dark to avoid oxidation of the compounds. The results were expressed as mg of malvidin-3-glucoside g^−1^ of fresh weight (FW).

### 3.5. Analysis of Total Phenolic Content by Folin-Ciocalteu Method

For the extraction of phenolic compounds, 0.25 g of skin tissue from non-treated and ethanol-treated table grapes stored at 0 °C and after 7 days at 20 °C were homogenized with 0.5 mL of a solution of methanol (1% HCl acidified)-water (*v/v*) and mixed for 60 min at room temperature (RT). The extracts were centrifuged at 10,000× *g* for 10 min and the supernatants were collected. The pellet was then homogenized with acetonitrile 70%, incubated for 60 min at RT, and centrifuged at 10,000× *g* at RT for 10 min. Supernatant was collected and combined with previous extract supernatants. The final volume was set to 1 mL with methanol 50%-acetonitrile 70%. The supernatants were stored at −20 °C. The content of total phenolic compounds in the extracts was determined by the Folin-Ciocalteu method [61] and expressed as mg of gallic acid equivalents g^−1^ FW.

### 3.6. Antioxidant Activities Measured by 2,2-Azino-Bis-3-Ethylbenzothiazoline-6-Sulfonic Acid (ABTS) and Ferric Reducing Antioxidant Power (FRAP) Methods

For the determination of the antioxidant activity of It 681–30 table grapes, the same extracts as for the determination of total phenolic content were used. ABTS and FRAP methods were performed according to Romero et al. [46]. 

### 3.7. Statistical Analysis

The software SPSS v23.0 (IBM) was used for the statistical analysis. The different data obtained were analyzed by ANOVA (one-way analysis of variance), and their means ± standard deviation were grouped in subsets by the Tukey-b test (*p* < 0.05). The relationship between expression data was described as the Pearson product-moment correlation coefficient (*r*), *p* < 0.01 or *p* < 0.05. 

## 4. Conclusions

The application of ethanol vapor treatments to It 681–30 table grapes reduced the deterioration of table grape quality during storage at 0 °C and after the 7-day shelf-life period at 20 °C. However, the total phenolic content and the antioxidant capacity seems not to play a role in the improvement of table grape quality by the ethanol treatment. By contrast, among the mechanisms triggered in It 681–30 table grapes to cope with low-temperature storage and shelf-life at 20 °C, the activation of *STS7* and *PRs* together with *PIP* gene expression could play an important role in controlling fungal attack and weight loss, respectively. The results from this work open an interesting research line in order to extend the postharvest storage in table grapes but further works would be necessary to unravel the mechanisms implicated in the effect of ethanol treatment.

## Figures and Tables

**Table 1:** Soluble solids content (SSC), titratable acidity (TA), pH, weight loss, total decay, and rachis browning index of It 681–30 table grapes non-treated and treated with ethanol for 49 days at 0 °C and after the shelf-life period (49 d + 7 d at 20 °C).

		Air		Ethanol	
	Freshly-Harvested	49 d 0 °C	49 d Air + 7 d 20 °C	49 d 0 °C	49 d + 7 d 20 °C
SSC (%)	19.2 ± 0.2 a	20.8 ± 0.1 b	21.4 ± 0.3 b	20.8 ± 0.4 b	20.5 ± 0.7 b
TA (% Tartaric Acid)	0.37 ± 0.04 a	0.41 ± 0.00 a	0.40 ± 0.00 a	0.41 ± 0.00 a	0.40 ± 0.00 a
Maturity Index (SSC/TA)	51.89	50.48	53.15	50.43	51.04
pH	3.82 ± 0.03 a	3.84 ± 0.01 a	3.84 ± 0.01 a	3.84 ± 0.01 a	3.80 ± 0.02 a
Weight Loss (%)	-	4.29 ± 0.32 b	7.89 ± 0.01 d	3.24 ± 0.09 a	6.61 ± 0.10 c
Total Decay (%)	-	11.8 ± 0.2 b	59.9 ± 0.6 d	4.0 ± 0.5 a	32.7 ± 0.7 c
Rachis Browning Index	-	3.50 ± 0.50 b	4.00 ± 0.00 b	2.50 ± 0.50 a	3.50 ± 0.57 b

Different letters in rows indicate significant differences using the Tukey-b test (*p* < 0.05).

**Table 2:** Pearson correlation between *VviPAL, VviCHS, VviSTS7, VviMYB13, VviMYB14*, and *VviMYB137* from the skin of non-treated and ethanol-treated It 681–30 table grapes stored at low temperature and after the shelf-life period.

	*VviPAL*	*VviCHS*	*VviSTS7*	*VviMYB13*	*VviMYB14*	*VviMYB137*
*VviPAL*	1	0.451 *	−0.577 **	0.030	0.073	−0.338
*VviCHS*	0.451 *	1	−0.440 *	0.250	−0.361	−0.107
*VviSTS7*	−0.577 **	−0.440 *	1	0.300	0.056	0.607 **
*VviMYB13*	0.030	0.250	0.300	1	0.584 **	−0.339
*VviMYB14*	0.073	−0.361	0.056	0.584 **	1	−0.701 **
*VviMYB137*	−0.338	−0.107	0.607 **	−0.339	−0.701 **	1

** Correlation is significant at the 0.01 level. * Correlation is significant at the 0.05 level.

**Table 3:** Pearson correlation between *Vcgns1, Vcchit1b, VviOsmo*, and *VviTL1* from the skin (S) or the pulp (P) of non-treated and ethanol-treated It 681–30 table grapes stored at low temperature and after the shelf-life period.

		*Vcgns1*	*Vcchit1b*	*VviOsmo*	*VviTL1*
*Vcgns1*	S	1	0.834 **	0.762 **	0.638 **
	P	1	0.827 **	0.834 **	0.790 **
*Vcchit1b*	S	0.834 **	1	0.878 **	0.648 **
	P	0.827 **	1	0.721 **	0.671 **
*VviOsmo*	S	0.762 **	0.878 **	1	0.634 **
	P	0.834 **	0.721 **	1	0.834 **
*VviTL1*	S	0.638 **	0.648 **	0.634 **	1
	P	0.790 **	0.671 **	0.834 **	1

** Correlation is significant at the 0.01 level.

**Table 4:** Pearson correlation between *aquaporins* from the skin (S) or the pulp (P) of non-treated and ethanol-treated It 681–30 table grapes stored at low temperature and after the shelf-life period.

		*VviPIP12*	*VviPIP13*	*VviPIP21*	*VviPIP22*
*VviPIP12*	S	1	0.579 **	0.554 **	0.406
	P	1	0.810 **	0.170	0.053
*VviPIP13*	S	0.579 **	1	0.497 *	0.580 **
	P	0.810 **	1	0.109	0.255
*VviPIP21*	S	0.554 **	0.497 *	1	0.451 *
	P	0.170	0.109	1	0.264
*VviPIP22*	S	0.406	0.580 **	0.451 *	1
	P	0.053	0.255	0.264	1

** Correlation is significant at the 0.01 level. * Correlation is significant at the 0.05 level.

## Data Availability

Not applicable.

## Supplementary Materials

The following are available online at https://www.mdpi.com/article/10.3390/ijms22158138/s1.
